# Supplementary material for: The Mitochondrial Small Heat Shock Protein HSP22 from Pea is a Thermosoluble Chaperone Prone to Co-Precipitate with Unfolding Client Proteins
Source: Int J Mol Sci. 2019 Dec 21;21(1):97. doi: 10.3390/ijms21010097 (PMC6981728; doi:10.3390/ijms21010097)
Supplement: Supplementary file 1 [file ijms-21-00097-s001.pdf]

SUPPLEMENTARY DATA

```
TaHSP16.9        ----------------------------------MSIVRRSNVFDPFADLWA-----DPF   21
PsHSP22          ------NTNAMRQYDQHSDDRNVDVYRHSFPRTRRDDLLLSDVFDPFSPPRSLSQVLNMV   54
AtHSP21          AQDQRENSIDVVQQGQQKGNQGSSVEKRPG-QRLTMDVSPFGLLDPLSPMRTMRQMLDTM   59
                                                      :   .::**::   :     : .

TaHSP16.9        DTFRSIVPAISGGGSETAAFANARMDW--KETPEAHVFKADLPGVKKEEVKVEVEDGNVL   79
PsHSP22          DLLTDNPVLS----------AASRRGWDARETEDALFLRLDMPGLGKEDVKISVEQNT-L   103
AtHSP21          DRMFEDTMPVSGRNRGGSGVSEIRAPWDIKEEEHEIKMRFDMPGLSKEDVKISVEDNV-L   118
                 * : .               .  *  *  :*  .   :: *:**: **:**:.**:.  *

TaHSP16.9        VVSGERTKEKEDKNDKWHRVERSSGKFVRRFRLLEDA-KVEEVKAGLENGVLTVTVPKAE   138
PsHSP22          TIKGEEGAK--ESEEKE----KSGRRFSSRIDLPEKLYKIDVIKAEMKNGVLKVTVPKMK   157
AtHSP21          VIKGEQKKE--DSDDSWS--GRSVSSYGTRLQLPDNC-EKDKIKAELKNGVLFITIPKTK   173
                 .:.**.  :  :.::.     :*   :  *: * :.  : : :** ::**** :*:** :

TaHSP16.9        VKKPEV-KAIQISG   151
PsHSP22          EEERNNVINVKVD-   170
AtHSP21          VERKVI--DVQIQ-   184
                  :.      :::.
```

Figure S1. Alignment of wheat cytosolic TaHSP16.9, Arabidopsis chloroplastic HSP21 (AtHSP21) and pea mitochondrial HSP22 (PsHSP22) amino-acid sequences. The mitochondrial targeting sequence were removed from the sequence of AtHSP21 and PsHSP22 precursor proteins. Alignments were performed by Clustal omega using default parameters. The alpha crystallin domain (ACD) is indicated in red (after Scharf et al., 2001).
***** conserved residues; **:** strong similarity of amino-acids; **.** weak similarity of amino-acids

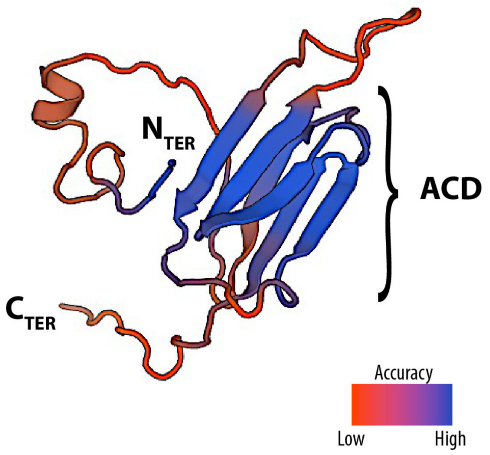

Figure S2. HSP22 3D structure modelling using Swiss Model. Template was the *Arabidopsis thaliana* chloroplastic HSP21 monomer (5nms.1.A in PDB database) : 0.76 % of coverage, QMean =-4,81, QMQE (Global Model Quality Estimation) = 0.58. The color scale (blue to red) indicates accuracy with the model (blue, regions with high accuracy; red, low accuracy). ACD = alpha crystallin domain.

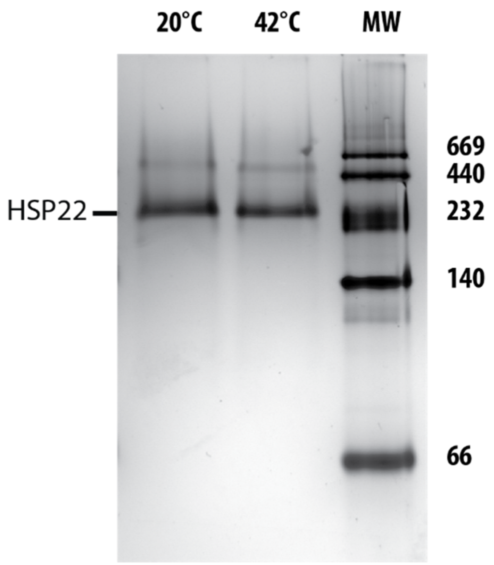

Figure S3. Analysis of heated and non-heated HSP22rec by native PAGE at 20 °C. Prior loading, proteins (5 µg) were either not heated (20°C) or heated for 20 min at 42 °C. Electrophoresis was carried out at 20°C on 10 % acrylamide gel. Molecular masses for the native markers (MW) are indicated in kDa.

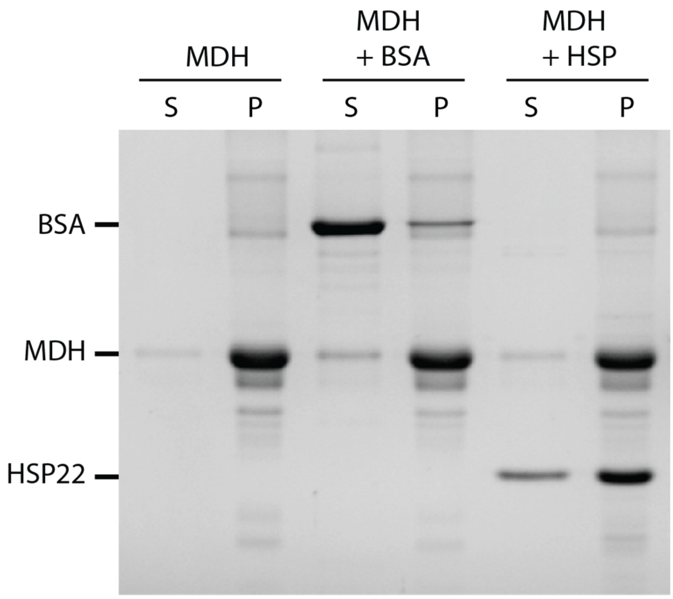

Figure S4. Thermal aggregation of malate dehydrogenase at 50 °C in the presence of thermostable proteins. Malate dehydrogenase (MDH) (10 µg) was heated 20 min at 50 °C alone or in the presence of bovine serum albumin (BSA) or of HSP22rec (HSP). Proteins were mixed together in a 1:1 mass ratio. After centrifugation, equal fraction volumes of supernatants and pellets were analyzed by SDS PAGE on a 12% acrylamide gel. Proteins were stained with colloidal blue.

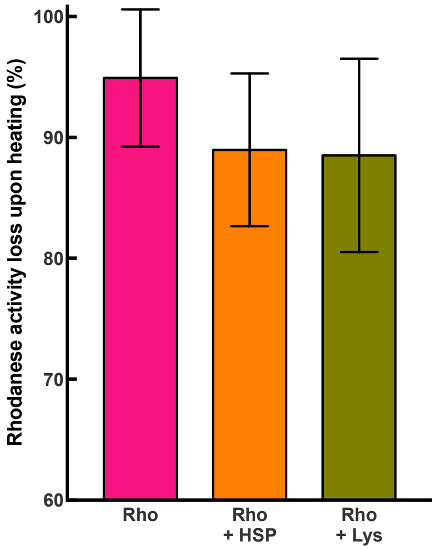

Figure S5: Loss of rhodanese activity when heated alone or in the presence of either HSP22 or lysozyme. Bovine liver rhodanese activity was measured alone (RHO) or in the presence of nine-fold molar excess of HSP22 (RHO+HSP) or lysozyme (RHO + Lys). Activities were determined for each condition, before and after heating at 50 °C for 5 min, as described in Material and Methods. The loss of rhodanese activity upon heating was calculated as followed : 100 x [(activity before heating minus activity after heating)/(activity before heating)]. Results are the mean of six independent experiments with three replicates each. No significant difference was found between the three conditions (p-value = 0.1345, Kruskal-Wallis test $p<0.05$)

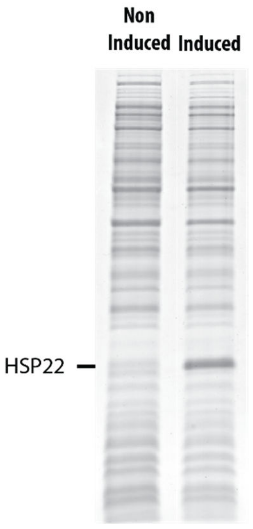

Figure S6. Over-expression of HSP22 in E. coli grown at 30 °C. *E. coli* B834(DE3)pLysS transformed with pET3a-HSP22 were grown at 30 °C until A_{600} reached 0.5. IPTG (0.4 mM) was added and cultured continued for 1h. Bacterial cells were recovered by centrifugation and suspended in SDS-PAGE sample buffer. Non-induced bacteria (NI) were used as a control. Total extracts from induced (I) and non-induced (NI) bacteria were analysed by SDS PAGE (13.5% polyacrylamide gel). Proteins were stained by colloidal blue.

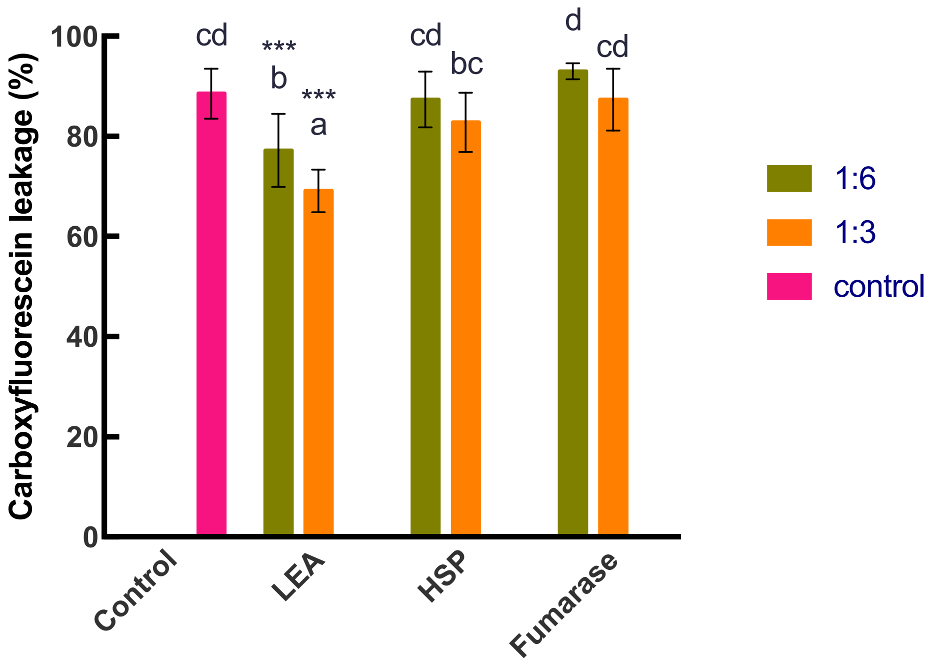

Figure S7. Effect of proteins (HSP22, LEAM, fumarase) on liposome integrity after drying and rehydration. 5 µg of POPC liposomes containing carboxyfluorescein (CF) were dried overnight alone (control) or in the presence of recombinant LEAM (LEA), recombinant HSP22 (HSP) or fumarase (Fumarase) at different protein:lipid mass ratio. After slow rehydration, the percentage of CF leakage was monitored by measuring the intensity of fluorescence. Results are the mean and standard deviation of three independent experiments with replicates (control, HSP22 and LEAM), and two independent experiments with replicates (Fumarase). Letters correspond to statistically different groups according to Anova and Tukey's test ($p<0.05$, n= 7 to 13). Significant differences with control are shown with asterisks (*** pvalue< 0.001).

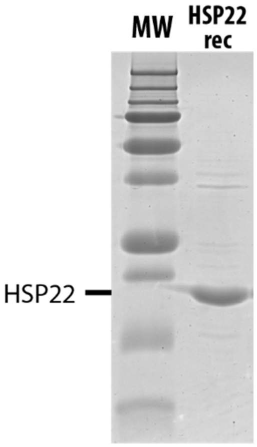

Figure S8. SDS PAGE analysis of the purified recombinant HSP22 (HSP22rec). HSP22rec was produced in *E. Coli* B834(DE3)pLysS and purified by chromatography as described in Materiel and Methods. The purified protein was analysed by SDS-PAGE (13.5 % polyacrylamide gel). MW = 250, 150, 100, 75, 50, 37, 25, 20, 15 kDa (from top to bottom). Proteins were stained by colloidal blue.

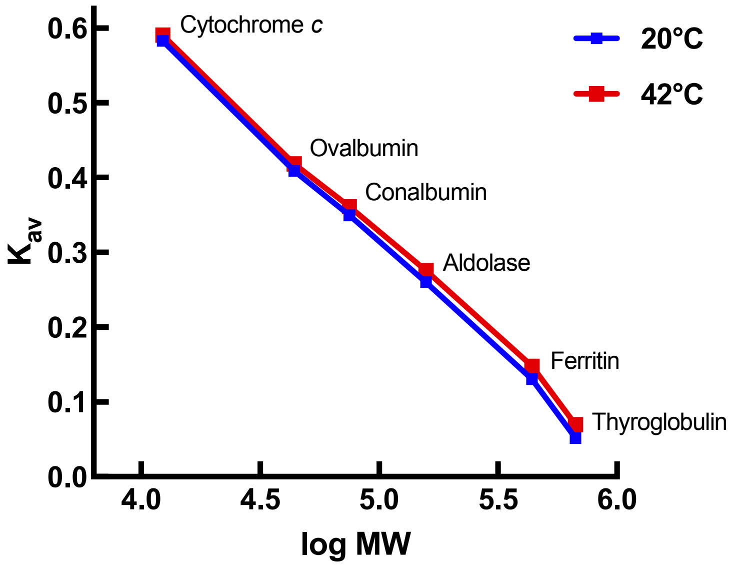

Figure S9. Calibration curves of the Superdex 200 10 /300GL (Ge Healthcare) column at 20 and 42 °C. Calibrations were performed using the gel filtration HMW calibration kit from GE Healthcare and 400 µg of cytochrome *c*. Dextran blue was used to determine the void volume.

MW = protein molecular mass in Da. K_{av} = partition coefficient = $(V_{elution} - V_{void})/(V_{column} - V_{void})$.
